# Efficacy of Large Language Models for Screening of Systematic Reviews on Periprosthetic Joint Infection

**DOI:** 10.3390/jcm15082830

**Published:** 2026-04-08

**Authors:** Woojin Shin, Jaeyoung Hong, Sunwoo Lee, Seongchan Park, Hyoungtae Kim, Suenghwan Jo

**Affiliations:** 1 College of Medicine, Chosun University, Gwangju 61452, Republic of Korea; woojin037@gmail.com (W.S.); jyhveritas@gmail.com (J.H.); doer2103@gmail.com (S.L.); tjdcks0605@gmail.com (S.P.); kht2769@naver.com (H.K.); 2 Department of Orthopedic Surgery, Chosun University Hospital, Gwangju 61453, Republic of Korea

**Keywords:** artificial intelligence assisted screening, large language model, periprosthetic joint infection, systematic review, total joint arthroplasty

## Abstract

**Background**: Periprosthetic joint infection (PJI) remains a devastating complication following arthroplasty. Systematic reviews of PJI provide essential evidence to inform clinical practice; however, the screening process remains labor-intensive. Recent advancements in large language models (LLMs) offer potential for automating literature screening, though evaluation of current generation models is needed. **Methods**: This validation study evaluated GPT-5, GPT-5 Pro, and Gemini 2.5 Pro in replicating the title/abstract and full-text screening stages of a published systematic review on intraosseous versus intravenous antibiotic prophylaxis in total joint arthroplasty. Title/abstract screening was performed on 165 articles, followed by a full-text eligibility assessment of 26 articles. Accuracy, sensitivity, specificity, and Cohen’s kappa (κ) were calculated against human screening decisions as the gold standard. **Results**: In title/abstract screening, GPT-5 Pro achieved the highest accuracy (92.1–92.7%) and specificity (98.6–99.3%), while GPT-5 demonstrated the highest sensitivity (84.6–96.1%). In full-text screening, Gemini 2.5 Pro showed the most consistent performance across repeated evaluations (κ = 0.839 in both trials), whereas GPT-5 Pro exhibited marked intra-model variability (κ = 0.399 to 0.920). **Conclusions**: Current-generation LLMs achieve near-human accuracy in systematic review screening for PJI research, though substantial intra-model variability underscores the continued need for human oversight in systematic review workflows.

## 1. Introduction

The exponential growth of orthopedic literature has established periprosthetic joint infection (PJI) as a primary focus of clinical research, yet the resulting surge in data has made the synthesis of evidence-based guidelines increasingly difficult. Systematic reviews of these studies provide essential evidence to inform clinical practice; however, the screening process for these reviews remains highly labor-intensive. Recently, artificial intelligence (AI)—particularly large language models (LLMs)—has advanced rapidly, gaining widespread attention for its transformative potential. In the medical field, LLMs have been applied across a broad spectrum of clinical and academic tasks, including automating medical documentation, supporting diagnostic and treatment decisions, and summarizing unstructured clinical data [1]. Beyond clinical care, LLMs are increasingly utilized in academic research to assist in drafting grant applications, improving manuscript clarity, and generating novel research questions [2]. The application of AI to systematic reviews (SRs), particularly during the screening stage, highlights its potential to significantly reduce the administrative burden on researchers [3,4,5]. The ability of LLMs to process massive datasets quickly and consistently has prompted investigations into whether these tools can reliably replace or augment human effort. Recent studies suggest that LLMs can substantially reduce reviewer workload while maintaining accuracy levels comparable to human reviewers [6]. However, significant variability in model performance remains a critical limitation, necessitating further rigorous evaluation.

Periprosthetic joint infection imposes a disproportionate burden on both patients and healthcare systems. The incidence of PJI following primary total joint arthroplasty ranges from approximately 0.5% to 1.2%, rising substantially following revision procedures [7]. Each episode typically necessitates complex, often multi-stage surgical intervention accompanied by prolonged antibiotic therapy, generating substantial costs and contributing to an estimated 23–25% of revision knee arthroplasty and 12–15% of revision procedures [7,8]. The resultant surge in PJI-related research has produced an extensive body of literature spanning diagnostic criteria, antibiotic prophylaxis strategies, surgical techniques, and novel adjunct therapies. Synthesizing this evidence through systematic reviews is essential for informing clinical guidelines and evidence-based practice; however, the rapidly expanding volume of publications renders manual screening an increasingly resource-intensive undertaking, frequently requiring hundreds of hours of reviewer effort per project [9,10].

While systematic reviews and meta-analyses are essential for guiding evidence-based clinical practice, the traditional process is notoriously time-consuming and labor-intensive [9]. The screening of titles, abstracts, and full texts often requires reviewers to manually assess hundreds or thousands of studies, frequently delaying the dissemination of critical findings [10]. Prior studies employing GPT-4-based models reported only modest results, with accuracies plateauing around 70% [3,4]. These findings underscored a gap between the theoretical potential of AI and its practical utility, highlighting the need for continuous reassessment as newer-generation models emerge. The subsequent development of GPT-5, GPT-5 Pro, and Gemini represents a substantial generational advance over GPT-4 based systems, with markedly improved reasoning, capability, contextual understanding, and instruction adherence [11,12]—providing strong rationale for updated evaluation in specialized clinical domains such as PJI.

Despite these advances, the applicability of LLM-assisted screening to specialized orthopedic domains such as PJI remains largely uncharacterized. Existing evaluations have predominantly focused on non-orthopedic subspecialties, where inclusion and exclusion criteria may differ substantially from those required for PJI research [3,4,5,6]. PJI systematic reviews are characterized by narrow PICO frameworks, domain-specific terminology (e.g., intraosseous versus intravenous antibiotic delivery), and a predominance of retrospective and prospective cohort designs rather than large randomized controlled trials—features that may challenge LLM screening performance in ways not captured by prior evaluations. Furthermore, the reproducibility of LLM outputs across repeated screening sessions has received limited attention, despite being a fundamental requirement for transparent and reliable evidence synthesis. The present study was therefore designed to address these gaps by rigorously evaluating the performance, consistency, and inter-trial reproducibility of current-generation LLMs in replicating the full screening workflow of a validated PJI systematic review.

Building on this context, the present study examines the efficacy of current-generation LLMs in performing the dual screening steps of a systematic review [13]. Our aim for the current study was to evaluate whether these advanced models can successfully navigate both title/abstract and full-text screening, thereby clarifying the current capabilities and limitations of AI-assisted methodology in the field of periprosthetic joint infection (PJI).

## 2. Material and Methods

This validation study evaluated the performance of three large language models (GPT-5, GPT-5 Pro; OpenAI, San Francisco, CA, USA, and Gemini 2.5 Pro; Google, Mountain View, CA, USA) [11,12] in the screening and eligibility assessment stages of a systematic review. In orthopedics, infection prevention in total joint arthroplasty is a critical issue, as periprosthetic joint infection remains one of the most serious complications following arthroplasty surgery [7]. Accordingly, the optimal route of antibiotic administration has been widely investigated, with intraosseous and intravenous delivery strategies being systematically compared in prior studies [13].

The systematic review conducted by Lee et al. [13], which directly compared these two antibiotic administration routes to manage PJI, was performed and was validated by peer review recently. Human screening decisions from Lee et al. [13] served as a reference standard for each stage.

Lee et al. [13] was selected as the reference standard for several reasons. First, it employs clearly defined PICO criteria, ensuring transparency and reproducibility of the evaluation. Second, it focuses specifically on PJI in the context of total joint arthroplasty, directly aligning with the clinical scope of the present study. Third, and most importantly, members of the present research team participated directly in its conduct, providing access to the identical article database used during the original human screening. This ensured that LLM screening decisions could be compared against the human reference standard on the exact same record set, maximizing the ecological validity of the evaluation.

### 2.1. Study Design

In the first stage, title and abstract screening was conducted on the retrieved articles from Lee et al. [13], and the decisions made by each LLM were compared against the human reference standard [13]. In the second stage, full-text eligibility screening was conducted on 26 articles that had been identified by the human reviewers after their initial title/abstract screening. Model performance was then evaluated by accuracy, sensitivity, specificity, and Cohen’s kappa. Because large language models may retain contextual traces from prior interactions or cached chat histories that could influence subsequent outputs, evaluations were repeated using separate model sessions to eliminate potential cross-session memory effects [14].

### 2.2. Prompt Entry into LLMs

Prompts for each step were designed to align closely with the criteria outlined by Lee et al. [13]. A structured prompt-engineering framework was applied, incorporating role prompting, step-by-step task instructions, and example-based formatting to ensure standardized screening decisions [15]. The exact prompt is provided in Appendix A.1.

For the title and abstract screening stage, prompts were designed to reflect the inclusion and exclusion criteria specified in the reference study by Lee et al. [13]. The inclusion criteria instructed the model to identify randomized controlled trials, prospective studies, and retrospective studies that reported clinical outcomes relevant to the predetermined PICO framework (Table 1). Conversely, the prompt directed the model to exclude manuscripts lacking original clinical data (such as commentaries, editorials, or expert opinions), studies unrelated to the PICO topic, laboratory-only or non-clinical investigations, studies involving patients with pre-existing infections, and review articles or systematic reviews.

The title and abstract for each paper were provided as text based on the dataset from Lee et al. [13]. Each title and abstract was entered into LLMs with the prompt that instructed the model to screen through the titles and abstracts that meet the criteria. Data that were extracted specific to this step of title/abstract screening included: study titles that both LLM and Lee et al. selected (true positive), study titles that both LLM and Lee et al. excluded (true negative), study titles that LLM selected but Lee et al. excluded (false positive), and study titles that LLM excluded but Lee et al. selected (false negative).

For the full-text screening stage, the prompts were designed to guide the model through a structured evaluation process. Studies were excluded if the patient population did not involve individuals undergoing arthroplasty (THA or TKA), or if intraosseous antibiotic administration during arthroplasty was not included as the intervention of interest. The model was further instructed to exclude studies unrelated to the research question, duplicates identified by matching titles or PMIDs, and publications that did not provide original clinical evidence (such as reviews, commentaries, descriptive reports, or news articles), unless statistical clinical outcomes were reported. Additional exclusion criteria included non-English publications, animal-only research, and studies lacking verification in a clinical setting. In cases where the eligibility of a study could not be confidently determined due to incomplete or unclear reporting, the model was instructed to label the study as unclear rather than force a classification. This stepwise screening ensured that only clinically relevant and methodologically appropriate studies were included for subsequent analysis. PDF files for the full text of each article were obtained for eligibility. For each article, the pdf file was uploaded to LLM with the prompt for eligibility. Data extracted at the title and abstract screening stage included four primary classification outcomes: studies selected by both the LLM and Lee et al. [13] (true positives), studies excluded by both sources (true negatives), studies selected by the LLM but excluded by Lee et al. [13] (false positives), and studies excluded by the LLM but selected by Lee et al. [13] (false negatives). In addition to these outcome classifications, the specific reasons for exclusion were also recorded based on the predefined screening criteria. For each exclusion decision, the corresponding exclusion category was recorded, including lack of original clinical data, absence of relevance to the PICO framework, non-clinical study design, inclusion of patients with pre-existing infections, or publication type that did not provide evaluable outcomes. This allowed for qualitative assessment of the types of errors made by the LLM and provided insight into systematic patterns of misclassification during the screening process. To assess intra-model reproducibility, the screening process was conducted twice for each model under identical conditions. The first trial was performed on 12 October 2025, and the second on 1 November 2025, with a three-week interval between sessions. Each trial was initiated in a freshly opened, independent model session to eliminate any potential cross-session memory or cache effects.

### 2.3. Outcomes of Interest

The primary outcomes were accuracy of LLMs in conducting title/abstract screening, and full-text screening. Secondary outcomes included sensitivity and specificity for both title/abstract screening and full-text screening.

### 2.4. Analysis

Accuracy was calculated as the sum of true positives and true negatives divided by the total number of screened studies. Sensitivity was defined as true positives divided by the sum of true positives and false negatives, while specificity was defined as true negatives divided by the sum of true negatives and false positives. These metrics were independently calculated for each screening stage, with true-positive and true-negative classifications defined relative to the human reviewer decisions from the reference study at both the title/abstract and full-text screening stages. All three models were evaluated on an identical article set—165 articles for title/abstract screening and 26 articles for full-text review—reflecting the same corpus assessed by human reviewers in Lee et al. [13], ensuring comparability both across models and relative to the original human screening process. Cohen’s kappa (κ) was additionally calculated to assess the degree of agreement between each LLM and the human gold standard, accounting for agreement expected by chance. Kappa values were interpreted as: <0.20, poor; 0.21–0.40, fair; 0.41–0.60, moderate; 0.61–0.80, substantial; and 0.81–1.00, almost perfect agreement [16].

### 2.5. Pilot Study and Feasibility Assessment

A pilot study was conducted to evaluate the feasibility of the screening workflow prior to the main analysis. The entire screening process was performed using two LLMs, GPT-5 and Gemini 2.5 Pro. The same evaluation criteria described in the Section 2.4 were used. As a result, GPT-5 and Gemini 2.5 Pro demonstrated accuracies of 90.3% and 89.0% in title/abstract screening. Both models achieved accuracies of 88.5% in full-text screening process. Detailed results are presented in Supplementary Table S1. These results were encouraging compared to previous studies and served as the basis for initiating the present study. Pilot study was conducted on 22 September 2025.

### 2.6. Generative AI Usage Statement

AI-assisted language editing was used to improve grammar, clarity, and readability; all scientific content, analyses, and final wording were reviewed and approved by the authors.

## 3. Results

### 3.1. Study Identification

**Figure 1:**
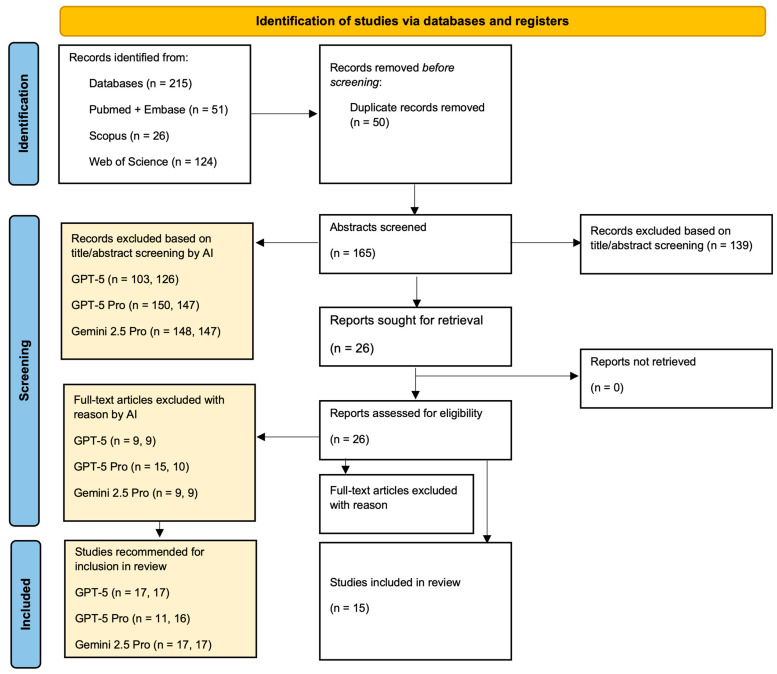
PRISMA flow diagram for the study selection process, with AI-driven screening pathways highlighted in colored boxes. For values presented in parentheses, the first value corresponds to the first trial and the second value to the second trial.

The PRISMA flow diagram is presented in Figure 1. A total of 215 records were identified from electronic databases, including PubMed and Embase (n = 51), Scopus (n = 26), Web of Science (n = 124), and the Cochrane Library (n = 14). After removing 50 duplicate records, 165 studies proceeded to title and abstract screening.

In the AI-assisted screening stage (highlighted in color within the flowchart), exclusion decisions varied between the first and second trials. During title and abstract screening, GPT-5 excluded 103 and 126 records in the first and second trials, respectively, while GPT-5 Pro excluded 150 and 147 records, and Gemini 2.5 Pro excluded 148 and 147 records. In comparison, conventional human screening excluded 139 records, resulting in 26 studies retrieved for full-text review.

During full-text screening, AI models again produced exclusion decisions, with GPT-5 excluding 9 studies in both trials, GPT-5 Pro excluding 15 and 10 studies, and Gemini 2.5 Pro excluding 9 studies in both trials. Human reviewers excluded 11 articles based on predefined PICO criteria. Ultimately, 15 studies were included in the final qualitative synthesis.

Among these, GPT-5 recommended 17 studies for inclusion in both trials, GPT-5 Pro recommended 11 and 16 studies, and Gemini 2.5 Pro recommended 17 studies in both trials, as illustrated in the AI-specific screening pathways in Figure 1.

### 3.2. Primary Outcomes

The primary outcome of this study was the accuracy of LLMs in reproducing human decisions during both title/abstract screening and full-text eligibility assessment. Table 2 describes all the specific performance.

In the title/abstract screening stage, averaged across two repeated screenings, GPT-5 achieved an accuracy of 82.1%, Gemini 2.5 Pro demonstrated 88.2%, and GPT-5 Pro showed the highest performance with 92.4%.

In the full-text eligibility screening stage, the models similarly demonstrated high accuracy. GPT-5 achieved an accuracy of 90.4%, Gemini 2.5 Pro achieved 92.3%, while GPT-5 Pro reached 82.7% when averaged across corresponding screenings. These eligibility results indicate that recently developed LLMs perform consistently across screening stages and achieve a level of reliability that approaches human screening in determining final study inclusion. Table 3 presents the gold standard studies included by each LLM.

Regarding intra-model consistency, Gemini 2.5 Pro demonstrated the most reproducible performance in full-text screening, producing identical results across both trials (κ = 0.839 in both trials, classified as almost perfect agreement). In contrast, GPT-5 Pro exhibited the greatest variability, with Cohen’s κ ranging from 0.399 in Trial 1 to 0.920 in Trial 2—a discrepancy that exceeded a two-category difference in agreement classification. GPT-5 also demonstrated notable variability in title/abstract screening, with κ increasing from 0.445 in Trial 1 to 0.602 in Trial 2.

To further characterize inter-trial reproducibility at the title and abstract screening stage, the overlap of exclusion decisions between the two trials was examined. Overlap rates were calculated as the proportion of concordant exclusion decisions relative to the total number of records excluded in either trial, with the union of both trial exclusion sets serving as the denominator. Among all records excluded in either trial, GPT-5 Pro demonstrated the highest exclusion overlap at 96.0% (144 of 150 records), followed by Gemini 2.5 Pro at 95.9% (142 of 148 records), and GPT-5 at 74.6% (94 of 126 records). These findings are consistent with the Cohen’s κ values observed for each model, with the lower overlap in GPT-5 reflecting its greater inter-trial variability in inclusion decisions.

Taken together, these findings show that modern LLMs demonstrate promising performance across both screening stages, supporting their potential role as effective supportive tools in systematic review workflows.

### 3.3. Secondary Outcomes

Secondary outcomes included the sensitivity and specificity of each LLM during both the title/abstract screening stage and the full-text eligibility assessment. Table 3 shows whether the 15 final studies included in Lee et al. were identified by each LLM.

During the title/abstract screening, GPT-5 demonstrated a sensitivity of 90.4% and a specificity of 80.6%. Gemini 2.5 Pro showed high specificity at 96.1%, although with a lower sensitivity of 46.1%. GPT-5 Pro achieved a balanced but conservative screening profile with a sensitivity of 57.7% and a specificity of 99%.

In the full-text eligibility screening, all models showed improved sensitivity relative to the title/abstract stage. GPT-5 achieved a sensitivity of 96.7% and a specificity of 77.3%, whereas Gemini 2.5 Pro reached 100% sensitivity with a specificity of 81.8%. GPT-5 Pro demonstrated a sensitivity of 80.0% and a specificity of 86.4%. These results highlight the varying tendencies of each model. GPT-5 showed relatively consistent performance across stages, whereas Gemini 2.5 Pro and GPT-5 Pro each showed a notable limitation in at least one of the title and abstract screening metrics.

## 4. Discussion

This study demonstrates that the newer generation of large language models (GPT-5, GPT-5 Pro, and Gemini 2.5 Pro) shows markedly improved performance in systematic review screening compared with earlier AI-based approaches in the field of PJI management. While similar studies have not been performed in the field of PJI, prior studies have reported accuracies near 70% [3,4], whereas the models evaluated in this study consistently achieved accuracies approaching 90% in both title/abstract and full-text screening. These improvements indicate that the performance has advanced substantially, increasing the applicability of LLM-assisted screening in real-world clinical research.

Despite these gains, the models displayed distinct performance profiles. GPT-5 demonstrated the most consistent overall performance across both screening stages, while Gemini 2.5 Pro and GPT-5 Pro each exhibited notable weaknesses in at least one title/abstract screening metric. These patterns highlight that LLM performance varies not only between models but also across screening stages, indicating that model selection can be a critical component of AI-assisted systematic review workflows.

Importantly, even the best-performing models demonstrated trade-offs between sensitivity and specificity. A highly sensitive model may select a larger number of irrelevant studies, increasing the volume of records requiring human review, whereas a model with high specificity risks excluding relevant studies and potentially compromising the completeness of the final review. These trade-offs highlight the need for a balanced screening strategy and indicate that fully automated decision making remains premature. Accordingly, the current findings support the role of LLMs as assistive tools capable of filtering large volumes of literature while retaining human oversight for final eligibility decisions.

Moreover, during full-text screening, all models were instructed to apply the same exclusion criteria defined by the human gold standard. However, instances were observed in which the LLMs excluded certain studies for reasons that differed from the human reviewers. Notably, when such discrepancies occurred, the LLMs often converged on the same alternative exclusion criteria across models. This pattern suggests that the screening process conducted by human reviewers may likewise be subject to potential bias.

A particularly notable finding was the substantial intra-model variability observed in GPT-5 Pro during full-text screening. Cohen’s κ fluctuated from 0.399 in Trial 1 to 0.920 in Trial 2, representing the largest within-model performance discrepancy observed across all models and screening stages. This likely reflects the stochastic nature of LLM outputs, wherein probabilistic text generation can produce divergent screening decisions even when identical prompts and inputs are applied [14,32]. Such non-determinism poses significant challenges for systematic review workflows, where reproducibility is a fundamental methodological requirement. In contrast, Gemini 2.5 Pro demonstrated the most consistent full-text screening performance, with identical results across both repeated evaluations (κ = 0.839), suggesting that model selection may be an important consideration when reproducibility is prioritized.

Together, these results illustrate ongoing improvements in LLM capabilities and the need for continued refinement before they can be deployed autonomously in clinical research environments.

From a practical standpoint, the complete LLM-assisted screening workflow—encompassing both title/abstract and full-text assessment—required approximately two hours per model, substantially less than the time investment typically required by conventional multi-reviewer human screening. The cost of deployment was limited to standard platform subscription fees, with no additional API or computational expenses. These figures suggest meaningful gains in efficiency when LLMs are used as a pre-screening tool. However, given the intra-model variability observed in the present study, a dual-trial approach may be considered for LLM-assisted screening. In this framework, cases with concordant decisions across independent trials may be accepted with greater confidence, while discordant results can be flagged for human adjudication. This strategy may help balance the efficiency gains of AI-assisted screening with the methodological rigor required for systematic evidence synthesis.

Beyond single-model strategies, the present data suggest that a multi-model union approach—in which a study is advanced if any of the evaluated models identifies it as eligible—may offer a potential means of mitigating individual model sensitivity limitations. In a post hoc analysis of the current dataset, when all three models were applied simultaneously using a union criterion, no missed studies were observed among the 15 final human-included studies at the full-text screening stage in either trial. At the title/abstract stage, one in Trial 1 and four in Trial 2 were missed, compared with 26 identified by human reviewers. Importantly, none of these corresponded to the final set of included studies, suggesting that they would likely have been excluded during full-text review regardless. Importantly, this approach does not rely on exclusion decisions from a single model; instead, it leverages complementary outputs across models, while human reviewers retain responsibility for verifying the smaller set of union-flagged inclusions. While these findings suggest that a multi-model union strategy may help reduce the risk of false negatives, this observation is dataset-specific and should be interpreted with caution. Given that this analysis was exploratory and not pre-specified, further validation in independent datasets and real-world screening settings is required. Nevertheless, these findings suggest that a structured multi-model workflow may represent a promising direction for improving sensitivity while maintaining scientific rigor.

### Limitations

This study has several limitations. First, model performance was evaluated using a single previously published systematic review in orthopedics. Although clinically relevant, the generalizability of results to other medical specialties or different screening tasks may be limited. Performance may vary substantially depending on methodological complexity, terminology, or complexity of inclusion and exclusion criteria.

Second, only the screening stages of the systematic review process were evaluated. Other essential components of systematic reviews, such as data extraction, risk-of-bias assessment, and evidence synthesis, were not examined. Therefore, the results reflect the feasibility of LLM-assisted screening only, not their performance across the full SR workflow.

Additionally, this study did not evaluate a fully automated pipeline in which LLMs performed title-and-abstract screening and subsequently processed only their own selected manuscripts for full-text review. While such a design would more closely simulate a real-world autonomous workflow, inter-model differences at the title-and-abstract stage would yield non-equivalent full-text inputs across models, thereby confounding direct performance comparisons at the full-text stage. To ensure fair and interpretable cross-model comparisons, full-text screening was therefore performed on the same set of human-selected manuscripts across all models. Future research could examine fully automated pipelines as a distinct research question, comparing their end-to-end performance against human-conducted workflows.

Third, although identical prompts and criteria were applied, repeated runs of the same evaluation produced inconsistencies in model decisions. This variability demonstrates the inconsistency of LLM outputs and raises concerns regarding reproducibility, which is critical for transparent and reliable evidence synthesis. Notably, GPT-5 Pro exhibited the most pronounced variability in full-text screening, with Cohen’s κ ranging from 0.399 to 0.920 across trials, whereas Gemini 2.5 Pro produced identical screening decisions in both repeated evaluations. Furthermore, differences in performance between the pilot study (September 2025) and the main study trials (October–November 2025) may partly reflect undisclosed model updates during this period, making it difficult to attribute all performance changes solely to stochastic variability.

Fourth, full-text screening relied on the model’s interpretation of PDF documents, which can be affected by formatting inconsistencies, OCR errors, incomplete extraction of methodological details, or misinterpretation of tables and figures. These challenges highlight areas where further methodological refinement or alternative approaches may help improve the reliability of AI-based full-text evaluation.

Fifth, although human screening was used as the gold standard, inter-reviewer disagreement within the original SR was not re-evaluated. Thus, model-to-human discrepancies may not always reflect true model error.

Lastly, our study explored the efficacy of LLMs in systematically reviewing the comparison of intraosseous and intravenous antibiotics for managing PJI, and results may vary depending on the subject.

## 5. Conclusions

The evaluated LLMs outperformed earlier AI tools in systematic review screening, achieving accuracies near 90% and demonstrating high specificity in title/abstract screening and strong sensitivity in full-text evaluation. These results illustrate meaningful progress toward reliable AI-assisted screening. Nevertheless, variability across models and inconsistent performance in certain metrics indicate that full automation remains premature.

At present, LLMs should be regarded as highly effective supportive tools capable of significantly reducing researcher workload, but they are not yet suitable for independent use in systematic review workflows. Continued refinements in model architecture, prompting strategies, and integration with SR platforms may eventually enable autonomous AI-driven screening, but human oversight remains essential for ensuring clinical reliability.

## Figures and Tables

**Table 1:** PICO framework from Lee et al. [13].

Population	Arthroplasty Patients (of Hip, Knee or Other Joints)
Intervention	Intraosseous antibiotics administered during arthroplasty.
Control	Patients undergoing arthroplasty, where only intravenous antibiotics were used
Outcomes	PJI incidence, systemic antibiotic levels, minimal inhibitory concentrations (MICs), local antibiotic concentrations achieved in soft tissues (or fat) and bone, and associated complications, including general as well as specific complications of vancomycin usage

**Table 2:** Comparison of performance metrics for title/abstract and full-text screening.

Metrics	GPT-5		GPT-5 Pro		Gemini 2.5 Pro	
	1st Trial	2nd Trial	1st Trial	2nd Trial	1st Trial	2nd Trial
Title/Abstract Screening						
Accuracy	76.9%	87.3%	92.1%	92.7%	88.4%	87.9%
Sensitivity	96.1%	84.6%	53.8%	61.5%	46.1%	46.1%
Specificity	73.4%	87.8%	99.3%	98.6%	96.4%	95.7%
Cohen’s κ	0.445	0.602	0.642	0.687	0.495	0.478
Full-text Screening						
Accuracy	96.2%	84.6%	69.2%	96.2%	92.3%	92.3%
Sensitivity	100%	93.3%	60%	100%	100%	100%
Specificity	81.8%	72.7%	81.8%	90.9%	81.8%	81.8%
Cohen’s κ	0.839	0.677	0.399	0.920	0.839	0.839

**Table 3:** Final included studies from Lee et al. [13] identified by each model.

Study	Year	Design	GPT-5		GPT-5 Pro		Gemini 2.5 Pro	
			1st Trial	2nd Trial	1st Trial	2nd Trial	1st Trial	2nd Trial
Young et al. [17]	2013	RCT	√	√	Excluded	√	√	√
Young et al. [18]	2014	RCT	√	√	√	√	√	√
Chin et al. [19]	2018	RCT	√	√	√	√	√	√
Young et al. [20]	2018	RCT	√	√	√	√	√	√
Harper et al. [21]	2020	Cohort ^R^	√	√	Excluded	√	√	√
Parkinson et al. [22]	2021	Cohort ^R^	√	√	Excluded	√	√	√
Klasan et al. [23]	2021	Cohort ^R^	√	√	√	√	√	√
Park et al. [24]	2021	Cohort ^R^	√	√	Excluded	√	√	√
Spangehl et al. [25]	2022	RCT	√	Excluded	Excluded	√	√	√
Harper et al. [26]	2023	RCT	√	√	√	√	√	√
Lachiewicz et al. [27]	2023	Cohort ^P^	√	√	√	√	√	√
Zhang et al. [28]	2024	RCT	√	√	√	√	√	√
Wininger et al. [29]	2024	RCT	√	√	Excluded	√	√	√
Christopher et al. [30]	2024	Cohort ^R^	√	√	√	√	√	√
McNamara et al. [31]	2025	Cohort ^R^	√	√	√	√	√	√

^R^ = retrospective study; ^P^ = prospective study.

## Data Availability

All data from this study are available on reasonable request to the corresponding author.

## Supplementary Materials

The following supporting information can be downloaded at: https://www.mdpi.com/article/10.3390/jcm15082830/s1, Table S1: Pilot Study Performance Metrics for Title/Abstract and Full-text Screening.

## Abbreviations

The following abbreviations are used in this manuscript:

AI	Artificial intelligence
LLM	Large language model
SR	Systematic Review
PICO	Population, intervention, comparison, outcome
PJI	Periprosthetic joint infection
THA	Total hip arthroplasty
TKA	Total knee arthroplasty
RCT	Randomized controlled trial
TP	True positive
TN	True negative
FP	False positive
FN	False negative
OCR	Optical character recognition

## Appendix A

### Appendix A.1

The following is the prompt used in title/abstract screening.

“

Role:

You are a research assistant helping with abstract screening for a systematic review. For each provided title and abstract, apply the PICO criteria as well as the inclusion and exclusion rules to decide eligibility.

PICO Criteria

- Population: Arthroplasty patients (hip, knee, or other joints)

- Intervention: Intraosseous antibiotics administered during arthroplasty

- Control: Arthroplasty patients receiving only intravenous antibiotics

- Outcomes:

  - Periprosthetic joint infection (PJI) incidence

  - Systemic antibiotic levels

  - Minimal inhibitory concentrations (MIC)

  - Local antibiotic concentrations (soft tissue, fat, bone)

  - Associated complications

Inclusion Criteria

- Randomized Controlled Trials (RCTs)

- Prospective studies

- Retrospective studies

- Must report data relevant to the above PICO

Exclusion Criteria

- Manuscripts without original clinical data (opinions, commentaries, editorials)

- Studies not related to the PICO topic

- Non-clinical, pre-clinical, or laboratory-only studies

- Studies involving patients with pre-existing infections

- Review papers or systematic reviews

Screening Procedure

1. Review each title and abstract in order.

2. Determine whether the study design meets the inclusion criteria.

3. Check whether the study population, intervention, and outcomes are relevant to the PICO.

4. Exclude if it clearly matches any exclusion criteria.

Output Format

For each abstract, respond in this structure:

[Number] Decision: INCLUDE/EXCLUDE

Reason: [1–2 sentence justification]

Input Example

1. Title: Example Study A

Abstract: [text here]

2. Title: Example Study B

Abstract: [text here]

Output Example

1. Decision: INCLUDE

Reason: Retrospective cohort in knee arthroplasty; intraosseous vs IV antibiotics compared; reports PJI outcomes.

2. Decision: EXCLUDE

Reason: Narrative review; no original clinical data.

“

### Appendix A.2

The following is the prompt used in full-text screening.

“

Role:

You are a research assistant performing eligibility screening for a systematic review.

Your task is to read the attached full-text PDF and determine eligibility based on predefined criteria.

 Exclusion Criteria (check in order):

1. Population: Exclude if patients are not arthroplasty patients (THA or TKA).

2. Intervention: Exclude if intraosseous (IO) antibiotics administered during arthroplasty are not used.

3. Irrelevant study: Exclude if the study is unrelated to the topic.

4. Duplicate results: Exclude if the PMID or title is a duplicate of an already included paper.

5. Publication type: Exclude if it is a review, commentary, case report, descriptive study, or news article.

  - If statistical outcomes are reported (cohort, RCT, retrospective), keep it.

6. Language: Exclude if the paper is non-English.

7. Non-human: Exclude if the study is animal-only.

8. Non-clinical: Exclude if findings are not verified in a clinical setting.

9. Unclear/Ambiguous: If the paper does not provide enough information to clearly determine eligibility, mark as **UNCLEAR**.

 Screening Procedure:

1. Read the full PDF carefully.

2. Check each criterion sequentially (1 to 9).

3. If a study fails any step, stop there and mark as EXCLUDE with the specific criterion number and reason.

4. If the study passes all 9 criteria, mark as INCLUDE.

5. If information is insufficient or ambiguous, mark as UNCLEAR and explain why.

 Output Format:

- Decision: INCLUDE/EXCLUDE/UNCLEAR

- Reason:

  - If EXCLUDE → state the exact step number and reason.

  - If INCLUDE → confirm it passed all 9 criteria.

  - If UNCLEAR → explain what was missing or ambiguous.

“
