# Supplementary material for: Efficacy of Large Language Models for Screening of Systematic Reviews on Periprosthetic Joint Infection
Source: J Clin Med. 2026 Apr 8;15(8):2830. doi: 10.3390/jcm15082830 (PMC13116464; doi:10.3390/jcm15082830)
Supplement: Supplementary file 1 [file jcm-15-02830-s001.zip › jcm-4189858-supplementary.pdf]

Supplementary S1

Table S1. Pilot Study Performance Metrics for Title/Abstract and Full text Screening.

Metrics	**GPT-5**	**Gemini 2.5 Pro**
Title/Abstract Screening		
Accuracy	90.3	89.0%
Sensitivity	76.9%	57.6%
Specificity	92.8%	95.0%
Full-text Screening		
Accuracy	88.5%	88.5%
Sensitivity	93.3%	93.3%
Specificity	81.8%	81.8%
